# Recent Advances in Honey-Based Nanoparticles for Wound Dressing: A Review

**DOI:** 10.3390/nano12152560

**Published:** 2022-07-26

**Authors:** Norfarina Bahari, Norhashila Hashim, Abdah Md Akim, Bernard Maringgal

**Affiliations:** 1 Department of Biological and Agricultural Engineering, Faculty of Engineering, Universiti Putra Malaysia, Serdang 43400, Selangor, Malaysia; gs60827@student.upm.edu.my; 2 Malaysian Agricultural Research and Development Institute (MARDI), Serdang 43400, Selangor, Malaysia; 3 SMART Farming Technology Research Centre (SFTRC), Faculty of Engineering, Universiti Putra Malaysia, Serdang 43400, Selangor, Malaysia; 4 Department of Biomedical Sciences, Faculty of Medicine and Health Sciences, Universiti Putra Malaysia, Serdang 43400, Selangor, Malaysia; abdah@upm.edu.my; 5 Faculty of Resource Science and Technology, Universiti Malaysia Sarawak, Kota Samarahan 94300, Sarawak, Malaysia; mbernard@unimas.my

**Keywords:** dressing, honey, nanoparticles, wound healing

## Abstract

Wounds with impaired healing, including delayed acute injuries and chronic injuries, generally fail to progress through normal healing stages. A deeper understanding of the biochemical processes involved in chronic wound cures is necessary to correct the microenvironmental imbalances in the wound treatment designs of products. The therapeutic benefits of honey, particularly its antimicrobial activity, make it a viable option for wound treatment in a variety of situations. Integration with nanotechnology has opened up new possibilities not only for wound healing but also for other medicinal applications. In this review, recent advances in honey-based nanoparticles for wound healing are discussed. This also covers the mechanism of the action of nanoparticles in the wound healing process and perspectives on the challenges and future trends of using honey-based nanoparticles. The underlying mechanisms of wound healing using honey are believed to be attributed to hydrogen peroxide, high osmolality, acidity, non-peroxide components, and phenols. Therefore, incorporating honey into various wound dressings has become a major trend due to the increasing demand for combination dressings in the global wound dressing market because these dressings contain two or more types of chemical and physical properties to ensure optimal functionality. At the same time, their multiple features (low cost, biocompatibility, and swelling index) and diverse fabrication methods (electrospun fibres, hydrogels, etc.) make them a popular choice among researchers.

## 1. Introduction

**Figure 1:**
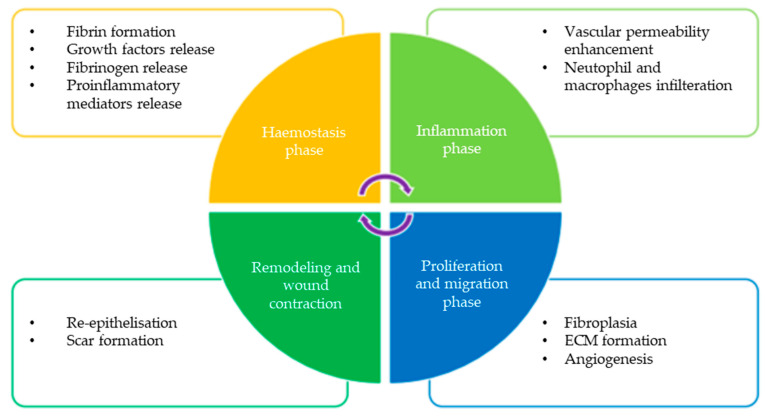
Wound healing process and the mediators/cells involved in the wound healing cascade. Reproduced from [8] with permission from Elsevier.

Wound healing has been seen for some time as a major therapeutic challenge that requires more effective wound therapy [1]. The wound healing process remains a complex clinical problem and has been thoroughly investigated in order to develop an ideal method for quick recovery while minimising scarring, which ensures the functions of preservation. Hamdan et al. reported that wound healing involves four stages: (i) coagulation and haemostasis; (ii) inflammation; (iii) proliferation; and (iv) wound remodelling (Figure 1) [2]. These stages occur at different timescales, in which initial coagulation can occur over minutes, while the process of tissue remodelling may last from several months to one year [3,4,5,6,7].

Interference with any of these four stages during wound healing can result in an impaired healing process [9]. This impaired healing is often a consequence of an underlying affliction (e.g., diabetes, age, and ischemia) or of external factors (e.g., infections) [3,10,11,12,13]. These systemic pre-conditioning situations frequently result in the partial or total prohibition of blood flow to a wound, disrupting the healing process by restricting the amount of nutrition, oxygen, waste elimination, and homeostasis available to the lesion [14].

Over the last several years, the field of research and development into wound dressing materials has evolved to a new level of standardisation, which appears to have led to a better knowledge of the aetiology of persistent wounds. Wound dressings exist in a variety of sizes and shapes, and they can be categorised on the basis of a variety of features. Firstly, wound dressings are divided into three types: traditional (e.g., gauze and gauze/cotton composites), biomaterial-based (e.g., allografts, tissue derivatives, and xenografts), and artificial (e.g., film, membrane, foam, gel, composites, and spray) wound dressings. Then, they can be classified into two categories: primary dressings, which are applied directly on the wound, and secondary dressings, which are used to cover the primary dressings [15,16].

A number of designed solutions for wound dressings with unique features and numerous functions have been developed to solve specific concerns with wound repair mechanisms. In order to achieve the proposed impact, the composition of wound dressings should meet the specified regulations of the respective product. The United States (US) Food and Drug Administration (FDA) regulates surgical and wound care dressings under the Medical Device Regulation Act and classifies them as Class I and Class II. Class I wound dressings are often associated with low-risk wounds, and their approval is subject to minimal regulatory requirements. Examples of dressings are detailed in Table 1.

Regardless of whether or not novel methods to facilitate wound healing can be established, wound management has found refuge in medicinal heritage and adopts some of the conventional therapies employed in the past. Honey is one of the traditional elements that act as powerful antimicrobial agents with a wide range of effects. The beneficial effects of honey on human health have long been recognised, and many of those benefits have been studied to determine how they work [18]. Various investigations into the composition and functional qualities of honey have been undertaken all over the world since the early twentieth century, and the results have been astounding. Numerous elements contribute to wound healing using honey, such as sugar, acid, hydrogen peroxide, and phenolic content. All of these elements are present in varying concentrations, depending on the source of the nectar, bee type, and storage [19]. Aside from sting bee honey, multiple studies on stingless bee honey for wound healing have also been conducted. Both sting bees (*Apis* spp.) and stingless bees (*Trigona* spp.) produce honey with excellent nutritional and medicinal value [20]. The production of honey by these bees has been widely used due to its biodegradable properties, as well as antimicrobial [21,22] and anti-inflammatory [23] effects.

In contrast to sting bee honey, stingless bee honey is distinguished by the fact that it is naturally stored in a pot (cerumen), which contributes to its therapeutic benefits, particularly in the wound healing process [21]. The author claimed that before nectars in cerumen pots are converted into honey, they must go through three distinct transformation stages. A huge portion of the water in the nectar evaporates, causing a physical change, which is the first metamorphosis. After that, there occurs a biological change that mostly consists of bacterial and yeast fermentation. These microbes come from an appropriate habitat that the bee has selected and work in symbiosis with the colony. The worker bees’ secretion of the enzymes needed to hydrolyse the nectar’s sucrose into fructose and glucose, as well as other chemicals, completes the chemical change. The water content, which is frequently higher than in other varieties of honey, is another notable similarity between stingless bee honey and other types of honey. The honey’s rich water environment encourages microorganisms to live there and prosper. The majority of these microbes are probiotics, and they secrete helpful enzymes that combine with those from stingless bees to improve and maintain the honey’s quality.

In line with the development of nanotechnology in the biomedical area, especially in wound management, nanoparticles have emerged as a viable therapy technique to expedite wound healing. In clinical practice, nanoparticles have been widely used in films, foams, hydrogels, hydrocolloids, contact layers, and multilayer dressings, in which the nanoparticle properties increase the probability of biological interaction and injury penetration [24,25]. Nanostructured materials have been used to stabilise and regulate the controlled release and delivery of natural and synthetic antimicrobials, so the effectiveness of such natural products (e.g., honey) has been improved, and the associated potential adverse events have decreased. As a result, embedding honey with nanomaterials is expected to produce certain characteristics that are relevant for applications such as wound dressing [5].

Thus, this review aims to highlight the fundamental elements of wound dressings, recent advancements in the application of honey-based nanoparticle wound dressings, and their future prospects. The review can serve as a primary resource for researchers, clinicians, and anyone else interested in honey-based nanoparticle technology and wound dressing in order to comprehend the potential and complexities of such wound healing.

## 2. Honey for Wound Healing

The usage of honey for wound healing purposes has been explored for centuries due to its antimicrobial, anti-inflammatory, and antioxidant properties. A variety of factors have been identified, including high sugar content, low water content, low pH, and the presence of endogenous hydrogen peroxide, which is attributed to the antimicrobial effects of honey. High sugar and other solutes in honey produce a strong osmotic gradient, which pulls fluid up the subdermal tissue. The high content of sugar in honey also provides an additional glucose source for cell proliferation (e.g., fibroblasts and endothelial cells) [26]. In addition, the sugar present in honey has been reported to interfere with bacterial quorum sensing, and recently, osmotic pressure has also been found to influence the bacterial capacity to form biofilms. The low water activity of honey leads to liquid fluid flows that remove bacteria, debris, and necrotic tissue from the wound and carry oxygen and nutrients from deep tissues to the area of the wound. Furthermore, the low pH of honey enhances tissue oxygenation but inhibits microbial growth [27].

**Figure 2:**
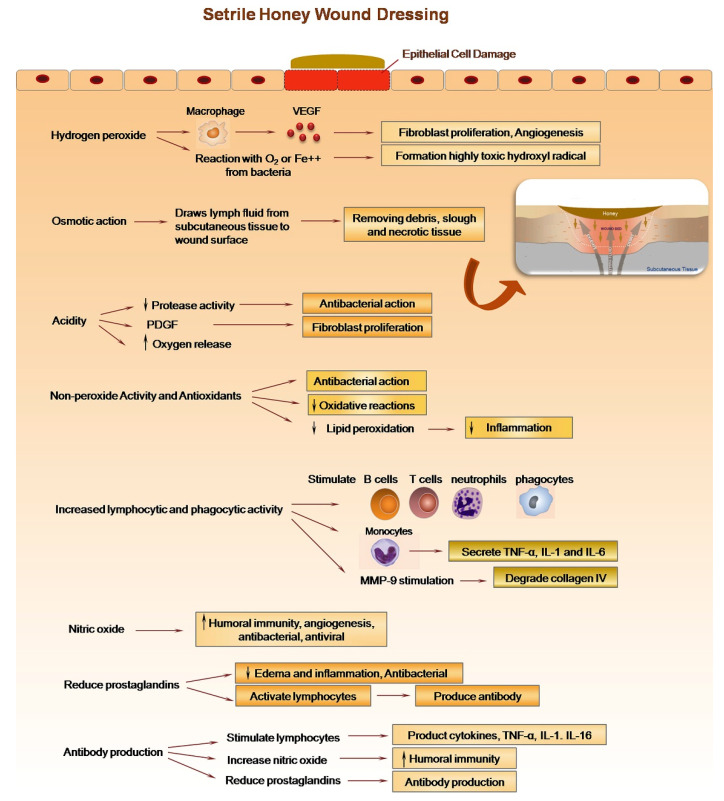
Mechanism of honey activity during wound healing. The effects of honey on the immune system, its debridement action, and its role in wound regeneration were boosted and stimulated by its antimicrobial, anti-inflammatory, and antioxidant activities, which significantly contributed to the wound healing process. Reproduced from [30] with permission from Elsevier.

Honey contains hydrogen peroxide (H_2_O_2_) that is produced when the enzyme glucose oxidase (added by honeybees to the nectar) is activated in a moderate dilution of honey and reacts with glucose, producing gluconic acid and H_2_O_2_ [28] (Figure 2). H_2_O_2_ is a disinfectant, as well as a powerful oxidiser. Several research studies have looked into the role of H_2_O_2_ in antibacterial activity, and they have found that removing H_2_O_2_ with catalase significantly diminishes the antibacterial power of the honey. Moreover, H_2_O_2_ is also antibacterial, which disinfects the wound site and stimulates the generation of the vascular endothelial growth factor [29].

Antioxidant capabilities are another medical property of honey that has been extensively explored. The presence of a range of phenolic compounds in natural honey contributes to its antioxidant effect. Phenolic compounds are a diverse group of secondary metabolites derived from plants in response to biotic and abiotic stresses, as well as oxidative damage. These compounds are transferred to honey via nectar. The phenolic compounds found in honey are classified into two groups, namely, phenolic acids and flavonoids. Phenolic acids and flavonoids scavenge free radicals, preventing tissue damage and regulating inflammation. In medicine, honey has been used in the treatment of surface wounds, burns, and inflammation and has a synergistic effect when applied with antibiotics [26]. Oryan et al. reported that the antioxidant effect depends on the relative positions of OH groups in the aromatic ring of phenolic acids, and gallic acid was likewise found to be the most powerful antioxidant among the other phenolic acid compounds [30].

**Figure 3:**
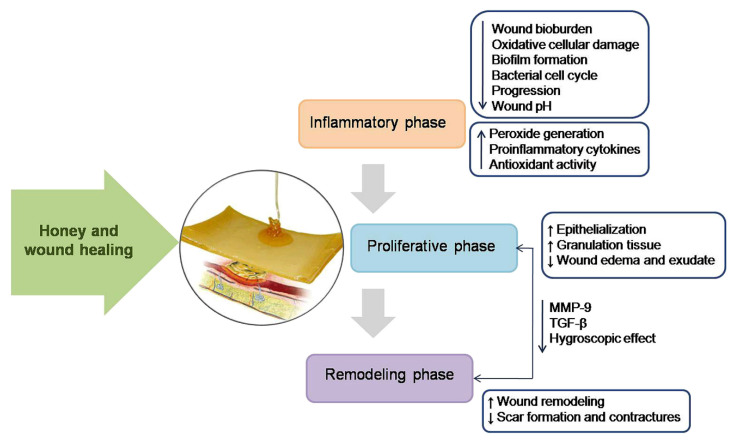
Modulatory impact of honey on the typical stages of wound healing. In the inflammatory phase, honey stimulates monocytes to release inflammatory cytokines, which play major roles in the initiation and amplification of inflammatory processes. It is able to remove debris and bacteria from the wound by stimulating neutrophils, macrophages, and phagocytosis during the inflammatory phase. The acidic nature of honey releases oxygen from the haemoglobin, a mechanism that stimulates granulation tissue formation and wound healing. It also promotes re-epithelialisation and holds the wound edges together due to its high osmotic pressure. Hydrogen peroxide contained at low levels in honey can stimulate the development of new capillaries and the growth and proliferation of fibroblasts and epithelial cells in wound tissue. Finally, in the remodelling phase, collagen is remodelled and realigned along tension lines, and cells that are no longer needed are removed by apoptosis. Reproduced from [30] with permission from Elsevier.

Several studies have reported the effectiveness of natural honey in wound healing and its ability to sterilise infected wounds, stimulate tissue growth, enhance epithelialisation, and minimise scar formation. The modulatory effect of honey on wound healing can be divided into three phases, i.e., the inflammatory phase, proliferative phase, and remodelling phase (Figure 3). Honey shows different effects in each phase and contributes to the process of wound healing [31]. Honey hinders bacterial placement, reduces pH, boosts antioxidant activity, enhances peroxide generation, and releases proinflammatory cytokines (TNF, IL-1, and IL-6) and PGE2 during the inflammatory phase. In the proliferative phase, it promotes epithelialisation and granulation and lowers oedema and exudate in the wound. Honey helps to reshape the wound and prevents scarring and contractures during the remodelling period. Honey has also been shown to boost MMP-9, TGF-β, and hygroscopic impact during the proliferative and remodelling phases [28].

Similar to sting bee honey, stingless bee honey has also been reported to have many medicinal properties, such as antiseptic, antimicrobial, anticancer, and anti-inflammatory effects, including wound-healing properties. Stingless bee honey is reported to have hundreds of bioactive compounds. Zulkhairi Amin et al. found that the honey of stingless bees has therapeutic properties that are similar to those of common sting bee honey, which prevents the growth of *Staphilococcus aureus* (*S. aureus*) with resistance to rifampicin and maintains *S. aureus* sensitivity to rifampicin in the treatment of the wound [20]. However, the composition and functional qualities of stingless bee honey vary depending on the source of the honey, which is determined by the location of the hive or the stingless bee species itself [32].

Furthermore, non-peroxide activity influences the antibacterial activity of stingless bee honey. According to Abd Jalil et al., it is uncommon to observe non-peroxide activity in A. mellifera honey [21]. To support their finding, they reported that the non-peroxide activity of stingless bee honey has a connection with the anatomical structure of the stingless bee itself. Further, the existence of catalase in the human body limits peroxide activity. This is due to the limitation of its counterpart. As a result of its desirable antibacterial characteristics, the application of stingless bee honey to the wounded area may reduce microbial infection and thus accelerate the healing process.

In addition, the antioxidant properties of stingless bee honey have a protective effect on wound healing. Previous research by Rosidi Sujanto et al. showed that protocatechuic acid, a strong antioxidant that can improve cell proliferation in the wound healing process, constitutes the major free phenolic acid in stingless honey [32]. Meanwhile, the results of analyses by Biluca et al. [23] and Maringgal et al. [33] revealed that stingless bee honey has a Folin–Ciocalteu reducing capacity, which is the ability to reduce ferric ions to ferrous ions (FRAP) and free radical scavenging activity (DPPH), thus implying a high antioxidant impact.

### Potential and Recent Commercial Application of Honey for Wound Healing

The promising and significant effects of honey for wound healing have prompted an increasing number of companies to produce honey-based wound dressings. Various honey-based products are available on the market, such as Activon^®^, L-Mesitran™, MediHoney^®^, Revamil^®^, and Principelle IF^®^. Each of these examples has been approved for medical use with different indications by the FDA [34], as summarised in Table 2.

Recently, the company “Advance Medical”, based in the United Kingdom (UK), invented the novel Activon^®^-Manuka Honey as an antimicrobial, debridement and de-sloughing, and malodour reduction wound dressing that is made entirely of Manuka Honey or a mixture of Manuka Honey and additives. The product can protect the wound bed by creating a moist wound-healing environment, which has been shown to promote faster healing. It is also suited for all types of wounds indicating a primary layer and has an antibacterial effect, including for cuts, abrasions, surgical intervention, leg ulcers, pressure ulcers, diabetic ulcers, and infected wounds. The dressing is designed to protect the wound, promote healing, and allow the passage of exudate [35].

A European Authorised Representative for Medical Devices named “Theo Manufacturing BV” has introduced the L-Mesitran™ wound dressing. The dressings are made up of different percentages of honey, as well as polyethylene, acrylic polymer gel, water, polyurethane film backing, and an adhesive border. Research findings by Lukanc et al. revealed that wounds with skin and subcutis involvement (type 1) healed in 14 to 49 days, whereas wounds in which skin, subcutis and muscles, tendons, and/or bones were involved (type 2) healed in 7 to 105 days when they were treated with L-Mesitran^®^ Soft medical honey wound gel. They also found that all wounds smaller than 10 cm^2^ healed in 7 to 56 days, while wounds larger than 10 cm^2^ healed in 28 to 105 days. Another interesting discovery was that odour and exudate in all wounds had dissipated by day 7, while the pH decreased from 7.6 to 7.0 in just 3 days [36].

A company with the name “Dermascience” in the US revealed their product named MediHoney^®^. The company claims to have solved a variety of wounds, ranging from minor abrasions, lacerations, and minor cuts to acute and chronic wounds such as leg/foot ulcers, infected wounds, odorous wounds, and surgical wounds. Furthermore, MediHoney^®^ has been shown to protect skin from friction and shear damage, prevent skin damage from frequent hand washing, and promote a moisture-balanced environment that is favourable to wound healing [37]. MediHoney^®^ Gel Wound & Burn Dressing contains 100% active Leptospermum honey in a hydrocolloid suspension. The FDA approved the product as non-toxic, natural, and safe.

Similarly, Hossain et al. reported Revamil^®^ Honey dressing as a potential and recent commercial application of honey for wound healing [37]. It has been found to heal a wide variety of wounds and skin conditions, including ulcers, dry and damaged skin, infected and chronic wounds, burns, and blistering. This dressing is suited for people of all ages, including diabetics. In addition, Revamil^®^ Honey dressings are intended for patients and practitioners, so they are designed to be simple to use and comfortable without compromising their effectiveness in wound healing [38]. Revamil^®^ Honey dressing is manufactured by Bfactory Health Products (Rhenen, The Netherlands) under standardised conditions in greenhouses from healthy bee colonies living in a controlled environment. By utilising this controlled production process, the company aims to provide reproducible wound healing and antibacterial activity in their Revamil^®^ Honey gel products.

**Table 2:** Commercially available honey-based wound dressings.

Commercially Available Honey-Based Wound Dressing	Composition	Application	References
Activon^®^	Manuka Honey or a mixture of Manuka Honey and additives	Protect the wound bed by creating a moist wound healing environment	[35]
L-Mesitran™	Mixture of different percentages of honey, polyethylene, acrylic polymer gel, water, polyurethane film backing, and an adhesive border	Promote healing	[36]
MediHoney^®^	Contains 100% active Leptospermum honey in a hydrocolloid suspension	Protect skin from friction and shear damage, prevent skin damage from frequent hand washing, and promote a moisture-balanced environment	[37]
Revamil^®^	Polyacetate sheet dressing impregnated with pure honey	Heal a wide variety of wounds and skin conditions, including ulcers, dry and damaged skin, infected and chronic wounds, burns, and blistering	[38]
Principelle IF^®^	Made up of an inert acetate substrate mixed with trace elements and a medical-grade honey ointment	Used to treat acute and chronic wounds (particularly those that are contaminated or infected), wounds that are moderately exuding, weakly exuding, or dry; it can also be utilised on wounds that are heavily exuding when a secondary dressing is used	[39]

Principelle IF^®^ is a sterile wound dressing made up of an inert acetate substrate mixed with trace elements and a medical-grade honey ointment. There are no synthetic ingredients in the dressing recipe. Medical-grade dark buckwheat honey is directly combined with minerals, trace elements, and oxides. Following the application of the dressing, the formulation is gradually released onto the wound surface, and gamma radiation is used to sterilise the products. The components are blended to an exceptionally high quality. The Principelle IF^®^ medical-grade honey wound dressing is used to treat acute and chronic wounds, particularly those that are contaminated or infected. It can be used on wounds that are moderately exuding, weakly exuding, or dry. It can also be utilised on wounds that are heavily exuding when a secondary dressing is used. Traditional dressings, a specific film dressing, foam or alginate dressing, or Principelle Matrix may be used to cover the wound, depending on the type of wound. The properties of Principelle IF^®^ make it easier to control the moisture content in the microenvironment of the wound and also the pH on the surface of the wound during the healing process. Principelle IF^®^ shields the wound from mechanical damage while allowing wound exudates to drain normally [39].

Overall, although all of these commercial dressings are claimed to be effective for treating (chronic) infected wounds, diabetic foot ulcers, decubitus, and burn wounds, scientific findings on these claims are limited or almost non-existent. Further scientific studies should be conducted to justify the various claims.

## 3. Application of Honey-Based Nanoparticles for Wound Dressings

**Figure 4:**
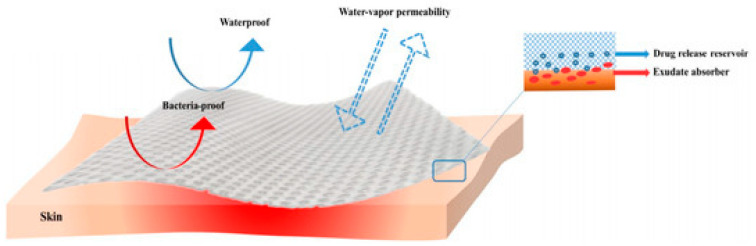
Properties of an ideal wound dressing. An optimal dressing is thus capable of preserving high humidity levels at the wound site while also eliminating excess exudates; in addition, it must be non-toxic, nonallergenic, comfortable, and cost-efficient, allow for oxygen and water vapour exchange, and protect against microbial invasion [25]. Reprinted from an open-access source.

Since ancient times, wound dressings have evolved from natural materials that merely covered and camouflaged the wound to modern materials today that are specifically engineered to deliver benefits. Wound dressings are technical products that can be classified by type, and each type of dressing will have one or more specific functions [40]. In their paper, Axibal and Brown explained that an ideal dressing provides pressure and haemostasis, provides site protection against infection and foreign material, immobilises surrounding tissues, prevents mechanical trauma, creates a moist healing environment, and wicks off blood and exudates (Figure 4) [41]. Moreover, an ideal wound dressing should be easy to apply and maintain, nonallergenic, aesthetically pleasing, cost-efficient, biocompatible, and readily stored [42]. With the growing demand, the development and fabrication of novel high-performance wound dressings have become a research focus in the field of medical materials [43].

### 3.1. Honey-Based Nanoparticles

Although honey has been used to heal wounds for thousands of years, there have been issues with applying honey to the wound bed since it drains out of the wound bed over time, causing discomfort to the patient [44]. The majority of clinical applications involve applying honey directly to the wound and covering it with gauze. This method of application is extremely inefficient because it necessitates multiple applications and dressing changes to keep the honey in the wound site.

**Figure 5:**
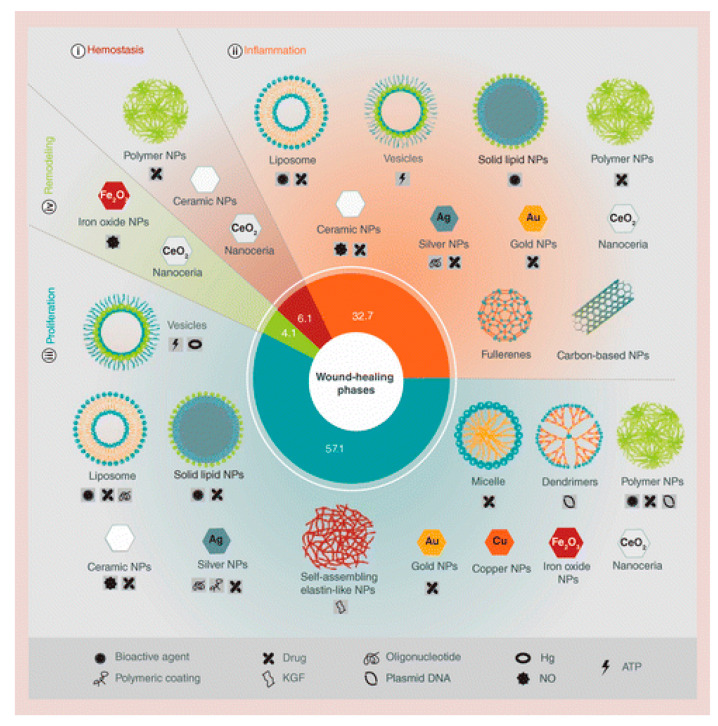
Different nanomaterials and their beneficial effects on the phase/s of wound healing. Republished with permission of Future Medicine Ltd. from [45].

**Figure 6:**
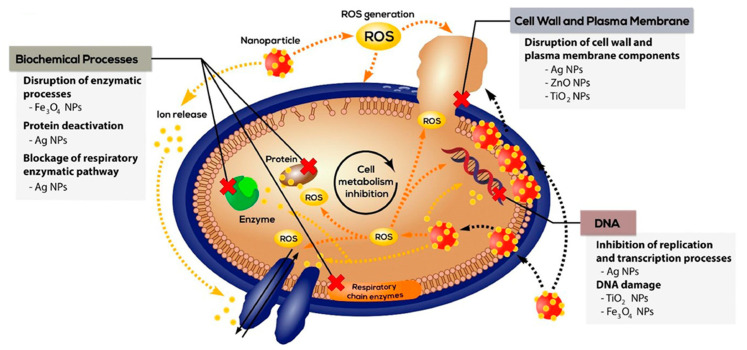
Various mechanisms suggested for the killing function of antibacterial nanoparticles [46]. Reprinted from an open-access source.

Meanwhile, according to Kalashnikova et al., various nanoparticles (NPs) have been found to be useful at various stages of wound healing [45]. NPs can be divided into four categories based on the stage of wound healing that they influence or correct (Figure 5). Based on the findings, most NPs improve wound healing by accelerating proliferation (phase III) and inflammation (phase II), whereas only a few NPs are known to aid in haemostasis and remodelling. Additionally, in another study, Homaeigohar and Boccaccini reported the various mechanisms suggested for the killing function of antibacterial NPs (Figure 6) [46]. However, NPs are rarely able to effectively resolve both wound healing and disinfection problems. As a result, they need to include bactericidal and healing-promoting functional agents. To overcome this problem, newer honey-impregnated dressings with NPs were introduced.

Honey-mediated green synthesis has been employed in recent years to synthesise gold NPs (Au-NPs), silver NPs (Ag-NPs), carbon NPs, platinum NPs (Pt-NPs), and palladium NPs (Pd-NPs). In most cases, honey acts as both a reducing and capping/stabilising agent and mainly acts as a precursor in the synthesis of NPs [47]. In general, the mechanism for the preparation of NPs using honey is as follows: First, the metal salt solution is dissociated to form the positive charge of the metal ions (Me^n+^), and then the metal ions react with the negative charge of the OH group of fructose and glucose in the honey to form [Me(honey)]^n+^. When sodium hydroxide (NaOH) is added, the reaction takes place and forms ([Me(OH)_n_/honey]), and after continuous stirring and ageing, metal or metal oxide NPs are formed.

Au-NPs are a popularly used nanomaterial in wound care, tissue regeneration, and targeted drug delivery for various biomedical applications. Au-NPs have been shown to exert antibacterial effects primarily by two mechanisms, as indicated by Data and Suresh Kumar [48]. As it enters the bacterial cell, an Au-NP inhibits the enzyme Adenosine triphosphate (ATP) synthase, lowering ATP levels and eventually leading to cell death due to a reduction in energy metabolism. Furthermore, Au-NPs are also effective by means of reactive oxygen species (ROS)-independent mechanisms against multiple drug-resistant organisms. A similar conclusion was reached by Naskar and Kim [49]. This is also consistent with the results found in a previous study conducted by Kalashnikova et al. [45]. A study performed by Philip in 2009 was one of the first articles on the green synthesis of Au-NPs with honey [50].

In 2010, Philip once again carried out the synthesis of NPs using honey. However, this time, the author produced Ag-NPs [51]. As reported previously, antibacterial Ag-NPs are among the most widely used NPs to treat burns and wound infections. According to Kalashnikova et al., the antibacterial activity of Ag-NPs is linked to physical parameters such as NPs’ shape and size [45]. The study confirmed that Ag-NPs improved the tensile qualities of restored skin by affecting collagen alignment. Another promising finding was reported by Mihai et al. [5]. They demonstrated that Ag-NPs coupled with tetracycline dramatically reduced the bacterial burden in both the superficial and deep tissue layers of a mouse model, resulting in faster recovery.

In addition to Ag-NPs and Au-NPs, studies have found that copper has a significant inhibitory effect on a variety of fungi and drug-resistant bacteria, which is very beneficial for chronic wounds in diabetes [45]. In 2019, Ismail et al. proposed a promising mechanism for the production of copper NPs (Cu-NPs) using honey. Copper ions (Cu^2+^) form during the reduction of copper nitrate solution and react with the negative charge on the hydroxyl group of fructose and glucose in honey to form [Cu(Honey)]^2+^. Upon the addition of NaOH, the reaction takes place, and [Cu(OH)_2_/honey] is formed. Cu-NPs are produced after the addition of ascorbic acid and the ultrasonic irradiation process [52].

Studies of the effect of the honey dosage on the synthesis of NPs have also been performed. Czernel et al. [53] demonstrated Ag-NPs synthesised using aqueous honey solutions at 2%, 10%, and 20% concentrations. The reaction was carried out at 35 °C and 70 °C. The presence and physicochemical properties of Ag-NPs were confirmed by analysis of the sample using ultraviolet–visible (UV-Vis) and fluorescence spectroscopy, scanning electron microscopy (SEM), and dynamic light scattering (DLS). The results show that the 20% honey solution caused the inhibition of the synthesis of NPs at 35 °C. The study also highlighted that the inhibition of Ag-NP synthesis in the 20% solution at 35 °C is related to the protein aggregation process. These results were verified by a resonance light scattering (RLS) instrument.

Furthermore, the effects of different honey concentrations at a fixed pH on the morphology of the Ag-NPs produced were previously reported by Haiza et al. [54]. Based on the study, they concluded that honey concentrations affect the particle size of the Ag-NPs produced. In an aqueous solution, a higher amount of honey tends to decrease the size of Ag^+^ compared to a lower amount of honey. It has been clearly proven that honey contains components such as fructose, glucose, various acids, vitamins, and minerals that act as both reducing agents and capping agents. It has been suggested that honey also contains other components, such as sucrose and proteins/enzymes, that may also promote Ag^+^ degradation. This is in agreement with the study by Rasouli et al. [55]. In that study, the easy and green synthesis of superparamagnetic iron oxide NPs (Fe_3_O_4_-NPs) was performed, and the particle size of the NPs was controlled by changing the concentration of the honey.

### 3.2. Application of Honey-Based Nanoparticles

**Figure 7:**
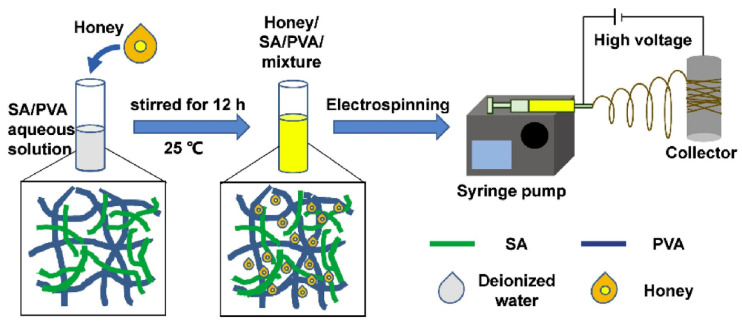
Schematic illustration of the solution preparation and electrospinning process. Reproduced from [57] with permission from Elsevier.

Recent advancements in honey-based nanoparticle applications for wound healing are summarised in Table 3. Honey-incorporated nanofibres (NFs) are gaining popularity as a result of their increased wound healing activity, which is achieved by combining the nanofibre structural benefits (Figure 7). In 2014, Arslan et al. investigated the effect of honey on the electrospinning process [56]. In their study, electrospinning was used to create fibrous mats from varying concentrations of polyethylene terephthalate (PET), PET/chitosan, PET/honey, and PET/chitosan/honey solutions. They reported that honey improved the morphology of chitosan-containing fibres, which previously exhibited a beaded or ribbon-like/branched morphology. In addition, PET/chitosan and PET/honey fibrous mats reached equilibrium water content in 15 min, and their absorption capabilities, which are critical for exudating wounds, ranged from 280% to 430% on a dry basis. Moreover, a cytotoxicity test revealed that the fibres had no toxicity activity.

The study of honey–chitosan NFs was continued by Al-Musawi et al. [58]. In that study, biocompatible antimicrobial nanofibrous wound bandages for topical skin treatments were prepared, characterised, and evaluated using honey/tripolyphosphate (TPP)/chitosan (HTCs) NFs loaded with capsaicin derived from the natural extract of hot pepper (*Capsicum annuum* L.) and loaded with Au-NPs. In comparison to HTCs and HTCs–capsaicin/Au-NP NFs and antibiotics, the results showed that HTCs–capsaicin and HTCs-Au-NPs were effective at reducing bacterial growth (*p* < 0.01). When compared to the untreated control, the MTT study demonstrated that the nanofibrous mats stimulated cell growth. In vivo tests revealed that the produced mats promoted wound closure rates more efficiently as compared to the control samples.

In consideration of the unique structural benefits of an electrospun NF membrane, as well as the superior material properties of alginate and honey in wound care applications, Tang et al. fabricated an electrospun honey/alginate/PVA nanofibrous membrane via electrospinning [57]. For this purpose, honey was incorporated into an alginate/PVA-based electrospun nanofibrous membrane to develop an efficient wound dressing material. They used scanning electron microscopy (SEM) and Fourier transform infrared spectroscopy (FTIR) to examine the shape and chemical content of the nanofibrous membrane, demonstrating that honey could be successfully incorporated into NFs. Moreover, they discovered that the antioxidant activity of the nanofibrous membranes increased as the honey content increased, implying the potential to limit the overproduction of reactive oxygen species. Using a disc diffusion assay and a dynamic contact assay, honey-loaded nanofibres were shown to have antibacterial action against Gram-positive *S. aureus* and Gram-negative *E. coli* bacteria. The biocompatibility and non-cytotoxicity of the nanofibrous membranes were demonstrated in a cytotoxicity test.

In 2021, Ghorbani et al. successfully developed honey-incorporated gum tragacanth (GT) NFs as a wound dressing mat [59]. Various methods were used to assess the physicochemical and biological properties of the produced NF mats. The bead-free and smooth NF mat with an EC/GT blending ratio of 85:15 was made by increasing the GT component in these NF mats. The influence of honey concentration on the various characteristics of the electrospun NFs was investigated. They discovered that increasing the honey concentration caused the NF diameter to rise from 335 ± 97 nm with 5% honey to 375 ± 120 nm with 20% honey. Another promising finding was that the honey concentration had a significant impact on NF swelling, with a high swelling degree recorded for EGH NFs with 5% honey content and a low swelling degree observed for NFs with 20% honey content. In addition, increasing the honey concentration in the EGH NFs increased the ability of the NFs to degrade. Moreover, the EGH NF mat with high honey content showed increased antioxidant activity and antibacterial capabilities, as well as better mechanical qualities and appropriate cell development, adhesion, and proliferation.

Hydrogels are another emerging method for utilising honey for wound dressing. Poly(vinyl alcohol) (PVA)/chitosan/honey/clay was developed and evaluated as a novel wound dressing by Noori et al. [60]. They studied the morphology and characteristics of produced nanocomposite hydrogels loaded with honey as a therapeutic model and then used X-ray diffractometry to confirm the exfoliated morphology of the nanocomposite. Swelling tests were performed at temperatures of 20 and 37 °C, as well as at different pH levels. The findings revealed that swelling increased as the temperature rose, with the maximum swelling occurring at a pH of 2. They stated that the MTT findings showed minimal cytotoxicity in the nanocomposite hydrogel system, and that the matching data revealed a faster honey release rate at higher pH values. Antibacterial activity for the proposed system was determined to be greater than 99%. Furthermore, the in vitro release of honey was investigated under similar circumstances, with the results confirming the established wound-healing capabilities of the system.

**Table 3:** Honey-based nanoparticle applications for wound dressing.

Intervention	Dressing Form	Findings	Reference
Honey-loaded ethylcellulose/gum tragacanth	Nanofibre	Good antioxidant abilities. High efficacy in preventing bacterial growth on the wound surface. Effective for promoting wound healing.	[59]
Honey/cellulose acetate	Nanofibre	Antioxidant properties. Effective for facilitating wound healing. Great efficacy in preventing bacterial growth on the wound surface.	[61]
Cellulose/honey composite	Composite sheet	The treated layer with three types of honey (*Thyme*, *Astragalus*, and *Ziziphus*) can absorb water 120 times faster than purified microbial cellulose.	[62]
Honey-loaded egg white/poly (vinyl alcohol)/clay	Hydrogel	The treated layer can collect exudate from te surface 1.37 times faster, which can accelerate wound healing by maintaining the moisture environment of the wound as well as preventing the penetration of bacteria into the wound. Decreased the swelling and dehydration capabilities by increasing the content of incorporated clay nanoparticles. Formed and maintained a moist area on the surface of infected and non-infected wounds. Accelerated the healing process of animal wounds.	[63]
Honey/chitosan/capsaicin/gold	Nanofibre	HTCs–capsaicin and HTCs-AuNPs are more effective at inhibiting bacterial growth than HTCs and HTCs–capsaicin/AuNP nanofibres and antibiotics. The nanofibrous mats increased cell growth compared to the untreated control. In vivo results show that the developed mats more effectively enhanced the rate of wound closure than the test samples. Has a relatively fast and efficient wound-healing ability.	[58]
Honey/PVA	Scaffold	Honey/PVA scaffold showed inhibition activity against proteases in chronic wounds, prevented bacterial infection from occurring over the wound site, and inhibited biofilm formation.	[64]
Erythromycin-releasing PVA/sucrose and PVA/honey	Hydrogel	The addition of honey improved the bio-adhesion of PVA/honey hydrogel compared to PVA/sucrose and pure PVA hydrogel. Antibacterial tests revealed that erythromycin-loaded PVA hydrogels inhibited the growth of *P. aeruginosa* and *S. aureus* bacteria.	[65]
Ag/alginate/honey	Hydrogel	The hydrogels exhibited strong bactericidal activity against the standard (at the total released silver concentration of ~9 μg/mL).	[66]
Honey/alginate/PVA	Nanofibre	Increased antioxidant activity, implying the ability to limit reactive oxygen species generation. Strong antibacterial activity against Gram-positive bacterium (*S. aureus*) and Gram-negative bacterium (*E. coli*). Non-cytotoxicity and biocompatibility.	[57]
Honey/iron oxide NPs	Cotton fabric	It had significant antibacterial properties against *E. coli*.	[67]
DNG/Ch/Manuka Honey	Composite sheet	Antibacterial studies confirmed >99% antibacterial activity against both *S. aureus* and *E. coli*. A bacterial adherence test showed that it was effective in preventing microbial invasion. Enhanced epithelialisation in terms of scar prevention and aesthetics.	[68]
Honey/PVA	Nanofibre	Scaffold with 1% honey exhibited antibacterial activity. Scaffold with 0.5% exhibited antioxidant and anti-inflammatory properties.	[69]
Silver/honey	Solution mixture	Increased the rate of healing in wounds infected by *P. aeruginosa*.	[70]
Poly (vinyl alcohol)/chitosan/honey/clay	Hydrogel	Higher pH values resulted in a faster honey release rate. MTT results revealed no cytotoxicity. Its antibacterial activity demonstrated that the suggested system has antibacterial activity of over 99%. The developed wound-healing capabilities of the system were proven in vivo.	[60]
Manuka Honey/silk fibroin	Silk Fibroin	Improved the antimicrobial activity of SF fibrous matrices, without negative effects on the excellent biocompatibility of SF. Good performance in improving wound healing.	[71]
Honey/chitosan hydrogel	Hydrogel	Honey plays a positive role in modulating wound healing when incorporated into a chitosan-based hydrogel matrix.	[72]
Honey/chitosan	Nanofibre	Improved wound healing compared to untreated controls, as evidenced by increased wound closure rates in mice.	[73]
PET/honey	Nanofibre	Cytotoxicity evaluation demonstrated that fibres exhibited no cytotoxic activity.	[56]
HA/PEO/Manuka Honey	Nanofibre	Promising results in terms of in vitro cell toxicity, antimicrobial effect, and antioxidant potential.	[74]

A quite similar study was conducted by Rafati et al. [63]. In that study, the authors demonstrated the use of honey-loaded bionanocomposite hydrogels based on egg white, PVA, and clay. They found that increasing the concentration of clay NPs reduced the swelling and dehydration characteristics of the developed wound hydrogel bionanocomposites. In the honey release test, they discovered that the presence of clay NPs was a critical element in honey release and produced control over the flow of honey from the wound dressings. A further novel finding was that the in vivo results of the honey-loaded bionanocomposite hydrogel demonstrated the excellent ability of the wound dressings to create and preserve a moist area on infected and non-infected wound surfaces and their ability to accelerate animal wound healing.

In the same year, Fathollahipour and colleagues investigated crosslinked PVA-based hydrogels containing honey and sucrose for erythromycin delivery [65]. Swelling measures demonstrated a reduction in hydrogel equilibrium swelling with the addition of sucrose and honey. The results demonstrated two things. First, the quantity of erythromycin released in the early hours for PVA/sucrose and PVA/honey was higher than the quantity released for PVA. Second, for PVA/honey hydrogel, the quantity of drug released was significantly reduced. This analysis found evidence that adding honey to PVA/honey hydrogel increased the bio-adhesion when compared to PVA/sucrose and pure PVA hydrogel. Furthermore, antibacterial testing revealed that erythromycin-loaded PVA hydrogels inhibited the growth of *P. aeruginosa* and *S. aureus* bacteria. Thus, they concluded that the hybrid hydrogels might be employed as functional wound dressings to control the rate of antibiotic distribution to the wound site and avoid infection.

The concurrent use of alginate hydrogels with Ag-NPs and honey components with the objective of targeting multidrug-resistant bacterial strains that cause nosocomial wound infections was reported by Stojkovska et al. [66]. They optimised a 5-month-long stable colloid solution, 50% honey, and ~8 nm Ag-NPs with a neutral pH in highly concentrated honey solutions for Ag-NP synthesis. The colloid solution was then utilised to make nanocomposite Ag/alginate hydrogels in various shapes (microbeads, microfibres, and discs) that retained all Ag-NPs as well as high fractions of honey components (40–60%), as assessed by the phenol–sulphuric acid and Folin–Ciocalteu techniques. The hydrogels were then characterised using UV–Vis spectroscopy and Fourier transform infrared-attenuated total reflectance spectroscopy, and their antibacterial activity was tested against a diverse range of Gram-negative and Gram-positive bacteria, including 13 multi-resistant clinical strains of *Acinetobacter baumannii* (*A. baumannii*), 1 clinical strain of *P. aeruginosa*, and 1 clinical strain of *S. aureus*. For the findings, they suggested that more results would be needed to support the bacterial resistance mechanisms, and, at the same time, tests on the potential of the novel Ag/alginate hydrogels with honey components should be carried out in detail in order to combat injuries and enhance healing as non-sticky, antibacterial, or bioactive dressings.

Neupane et al. investigated the antimicrobial activity of Himalayan honey-loaded Fe_3_O_4_ NPs with respect to both Gram-positive and Gram-negative bacterial strains of *S. aureus* and *E. coli* using the well diffusion method [67]. Himalayan honey-loaded Fe_3_O_4_ NPs were synthesised and characterised using X-ray diffraction (XRD) and SEM. The presence of honey on nanoparticles was confirmed by UV-Vis spectra. The antioxidant activity of these nanoparticles was studied against the 1, 1-diphenyl-2-picrylhydrazyl (DPPH) radical system. From XRD analysis, they reported that the average particle size was found to be 33–40 nm, and a needle-shaped porous structure of Himalayan honey-loaded Fe_3_O_4_ NPs was discovered from the SEM images compared to free Fe_3_O_4_ NPs, indicating the surfactant-like behaviour of the honey. In the DPPH radical system, the scavenging activities of Himalayan honey, free Fe_3_O_4_ NPs, and Himalayan honey-loaded Fe_3_O_4_ NPs ranged from 7.93–35.99%, 11.02–52.02%, and 16.10–80.52%, respectively, with corresponding IC50 values of 1.36 mg/mL, 1.09 mg/mL, and 0.52 mg/mL. For both bacterial strains, the antibacterial properties of all test materials demonstrated notable inhibition. In comparison to free Fe_3_O_4_ NPs, they discovered that the Himalayan honey and Himalayan honey-loaded Fe_3_O_4_ NPs had considerable antibacterial action against *E. coli*. As a result, they confirmed that Himalayan honey-loaded Fe_3_O_4_ NPs could be a promising antioxidant and antibacterial alternative.

**Figure 8:**
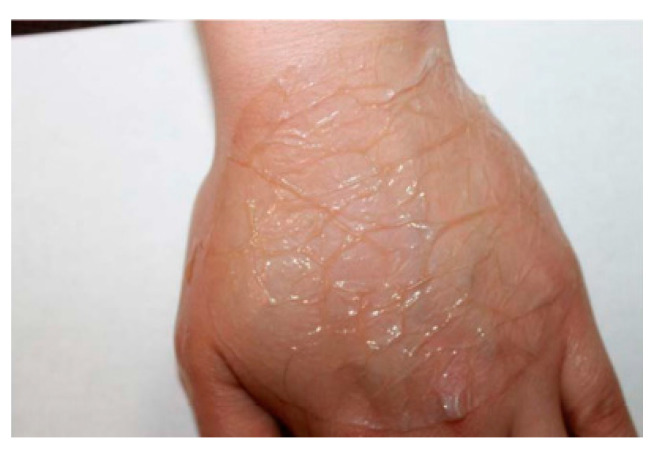
Transparency of microbial cellulose/honey sheet [62]. Reprinted from open source.

Considering its outstanding physical and chemical qualities, including high mechanical strength, high water absorption, and biodegradability, nano microbial cellulose (NMC) has attracted a lot of attention for its use as one of the important biomaterials in the medical industry. In a recent study, Meftahi et al. explored a nano microbial cellulose/honey composite for medical application, i.e., wound healing. At the outset of their research, three key features of Thyme, Astragalus, and Ziziphus honey were evaluated: pH, total soluble solids, and hydrogen peroxide content [62]. In the second stage, the zone of inhibition diameters of *E. coli* and *S. aureus* were measured. At the same time, ATR-FTIR, XRD, and SEM were used to investigate the chemical, physical, and surface morphology of the NMC pellicle. In addition, the air permeability and wettability of the samples were investigated in their research. From the findings, despite having the least amount of hydrogen peroxide, Thyme honey had the highest antibacterial properties. Furthermore, NMC treated with Thyme honey had 43% higher wettability and 49% faster sinking time than untreated NMC, and purified dry raw cellulose had 11% higher air permeability than dry raw cellulose at 400 Pa. Therefore, they concluded that the Thyme honey-treated NMC had a high potential for use in the medical field as a novel wound dressing (Figure 8).

Another promising study was conducted by [74]. In the study, the new Hyaluronic Acid/Polyethylene Oxide Nanofibres (HA-PEO-NFs), which have different inglobated active components (ACs), such as Manuka Honey, propolis, *Calendula officinalis*, insulin, and L-arginine, were prepared and characterised in terms of physicochemical and structural features, including morphology, fibre diameters, FTIR spectra, and Water Vapour Transmission Rate (WVTR). From the results, they found that all designed HA-PEO-ACs NFs displayed promising results with respect to in vitro cell toxicity, antimicrobial effect, and antioxidant potential.

Generally, these basic findings are consistent with research showing that integrating honey dressing materials and nanotechnology products under perfect conditions enhances the effectiveness of honey in wound healing. With all of these promising results, more research is needed to give full scientific support for its use, as well as to dispel any doubts and false acclaims.

## 4. Future Perspectives and Conclusions

The demand for advanced wound dressings is growing as a result of technological advancements, an increase in the number of surgical procedures, and the global prevalence of chronic wounds. The rising prevalence of chronic diseases such as diabetes, cancer, and autoimmune diseases is driving up demand for advanced wound dressing. Furthermore, from 2018 to 2026, the global antimicrobial wound dressing market is expected to be fuelled by an increase in demand for innovative dressings and new product introductions by market players. However, the relatively high costs of antimicrobial dressings are a major impediment to the growth of the global market. The global market for antimicrobial wound dressings can be divided into four segments: type, indication, end-user, and region [75].

Incorporating honey into various wound dressings has become a major trend due to the increasing demand for combination dressings in the global wound dressing market. Numerous studies have been conducted in recent years for honey-based nanoparticle wound dressings incorporated with nanomaterials such as gold, silver, chitosan, cellulose, and PVA. The main drawback for honey-based nanoparticles is the critical requirement to define the molecules in honey that are responsible for metal reduction as well as to identify which of those available honey formulations is suitable for producing nanoparticles. Additionally, there is also a critical need to understand the proper honey selection for usage in nanomaterials and wound dressings, as well as to identify the most prevalent honey proteins responsible for the stabilisation of metal ions in nanoparticle formation.

Although the efficiency of honey against bacteria has been maintained in designed wound dressings containing honey, more observations are urgently needed to corroborate its behaviour in an in vivo environment. To obtain a prolonged release and increase the likelihood of attaining long-term antimicrobial results, other honey-encapsulating techniques should be investigated.

## Figures and Tables

**Table 1:** List of dressings and associated level of risk [17]. Adapted from open-access source.

Type	Examples	Level of Risk	FDA Classification	Regulatory Requirements
Fabric dressings	Hydrophilic wound dressings, occlusive wound dressings, and hydrogel wound dressings	Low risk	Class I	Approval not required; the FDA only needs to be informed before marketing. It is the responsibility of the manufacturer to maintain the safety and quality of the product.
Advanced wound care dressings	Medihoney, Prisma, and Oasis wound matrix	Intermediate risk	Class II	510 (k) approval is required.

## Data Availability

Not applicable.
